# Trifecta achievement in patients undergoing partial nephrectomy: a systematic review and meta-analysis of predictive factors

**DOI:** 10.1590/S1677-5538.IBJU.2021.0095

**Published:** 2021-04-20

**Authors:** Nigemutu Bai, Muge Qi, Dan Shan, Suo Liu, Ta Na, Liang Chen

**Affiliations:** 1 Department of Mongolian Medicine Urology Affiliated Hospital Inner Mongolia University for Nationalities Tongliao China Department of Mongolian Medicine Urology, Affiliated Hospital of Inner Mongolia University for Nationalities, Tongliao,China;; 2 Department of Mongolian Medicine Gastroenterology Affiliated Hospital Inner Mongolia University for Nationalities Tongliao China Department of Mongolian Medicine Gastroenterology, Affiliated Hospital of Inner Mongolia University for Nationalities, Tongliao,China;; 3 Department of Mongolian Medicine Cardiology Affiliated Hospital Inner Mongolia University for Nationalities Tongliao China Department of Mongolian Medicine Cardiology, Affiliated Hospital of Inner Mongolia University for Nationalities, Tongliao,China;

**Keywords:** Kidney Neoplasms, Nephrectomy, Systematic Review [Publication Type]

## Abstract

**Purpose:**

The predictors of trifecta achievement in partial nephrectomy (PN) were poorly inquired and remained a controversial area of discovery. To evaluate predictive factors of trifecta achievement in patients undergoing PN.

**Materials and Methods:**

A systematic literature search was performed to identify relevant articles. Only studies focusing on postoperative trifecta achievement and exploring its predictor with multivariable analyses were included. The trifecta achievement was defined as negative surgical margins, warm ischemia time <25 minutes, and no complications. Merged odds ratio (OR) and 95% confidence interval (CI) were used to evaluate the predictive effect.

**Results:**

Thirteen studies with 7066 patients meeting the inclusion criteria were included. The rate of trifecta achievement ranged from 43.3% to 78.6%. Merged results showed that preoperative eGFR (OR: 1.01, 95% CI: 1.00, 1.02, P=0.02), operative time (OR: 0.99, 95% CI: 0.99, 1.00, P=0.02), estimated blood loss (OR: 1.00, 95% CI: 1.00, 1.00, P <0.001), tumor size (OR: 0.70, 95% CI: 0.58, 0.84, P <0.001), medium (OR: 0.39, 95% CI: 0.18, 0.84, P=0.02) and high PADUA score (OR: 0.23, 95% CI: 0.08, 0.64, P=0.005) were independently associated with trifecta achievement. A publication bias was identified for tumor size. Sensitivity analysis confirmed the stability of result for tumor size.

**Conclusions:**

Larger tumor size, medium and high PADUA score are associated with decreased probability of trifecta achievement. After verifying by further high-quality studies, these variables can be incorporated into tools to predict probability of trifecta achievement during clinical practice.

## INTRODUCTION

In 2020, kidney and renal pelvis cancer was estimated to be associated with nearly 73.750 newly diagnosed patients and 14.830 cancer-related deaths in the United States ( 1 ). Renal cell carcinoma accounts for the vast majority of these cases. Compared with radical nephrectomy, partial nephrectomy (PN) is more effective for cT1a renal masses in terms of surgically related mortality, overall survival, and renal function, and has become a standard treatment regimen ( 2 , 3 ). In addition, for larger renal masses (cT1b and cT2), a recent study has shown that PN can offer the same cancer control, better preserved renal function, acceptable surgical morbidity, and potential better long-term survival compared to radical surgery ( 4 ). With the development of medical instruments, PN has evolved from open surgery to laparoscopic and robot-assisted surgery, and became widely applied in managing highly complex kidney cancer ( 5 , 6 ).

As a novel concept from radical prostatectomy, the trifecta outcome was initially proposed by Hung et al. to describe the outcome of partial nephrectomy ( 7 ). It provides a definition of an ideal surgical outcome that includes the following three criteria: negative surgical margins, maximum renal function retention and patient recovery without complications. The use of trifecta rate as a key indicator of partial nephrectomy success has been widely reported ( 8 - 11 ). Recently, some researchers have proposed several anatomic classification scoring systems to classify and stratify patients into different anatomic complexity groups, and allow doctors to evaluate perioperative outcomes ( 12 - 15 ). In addition to these anatomic scoring systems, some other perioperative variables such as age, gender, BMI, tumor size, operative time, estimated blood loss have been studied as predictive factors for trifecta achievement in patients undergoing partial nephrectomy ( 16 - 20 ). However, inconsistent results reported by different studies confuse our understanding and interpretation. Hence, based on studies reporting predictive factors for trifecta achievement, we merged the results using the method of systematic review and meta-analysis.

## MATERIALS AND METHODS

The protocol of the present study was registered on PROSPERO (ID: CRD42020220307). The PRISMA checklist was presented in supplementary data.

### Literature researching

After establishing a prior study protocol, two authors independently used PubMed, Embase and Cochrane Library, respectively, to conduct a literature search for post-PN trifecta achievement until September 2020. The free-text strategy was considered best suited to this purpose: “post-PN trifecta achievement”. The key words included “partial nephrectomy”, “nephron sparing surgery”, “trifecta”, “trifecta achievement”. The language was restricted to English, non-English articles were filtrated. Publication type was restricted to original article, reviews, congress abstracts, letters to editor, editorials, erratum, and short communications were filtrated.

### Study selection

The studies focused on patients with renal tumor who had undergone partial nephrectomy and achieved trifecta or not. The trifecta achievement was defined as negative surgical margins, warm ischemia time <25 minutes, and no complications. Predictive factors of post-PN trifecta achievement were studied with multivariable logistic analyses and reported in included studies. The abstract of each study was evaluated to assess the eligibility of the study. Those studies that provided relevant data were chosen for detailed checking.

The studies were excluded due to the following reasons: ( 1 ) didn’t reported relevant outcomes, ( 2 ) without results from multivariable analysis, ( 3 ) inconsistent definition of trifecta achievement, ( 4 ) duplicated publication.

### Data extraction

Based on the included studies, the following data were extracted: ( 1 ) study features (first author’s name, publication year, study design, patient resource, study period, country, sample size); ( 2 ) patient characteristics (age, surgical procedure, T stage, rate of trifecta, variables included in multivariable analysis); ( 3 ) predictors of trifecta achievement (multivariable odds ratio [OR] and 95% confidence interval [CI] of age, body mass index (BMI), Charlson comorbidity index, preoperative estimated glomerular ﬁltration rate (eGFR), operative time, estimated blood loss, tumor size, N score component, RENAL score, PADUA score (medium or high vs. low)).

### Study quality assessment

For non-randomized controlled studies, the Newcastle-Ottawa Assessment Scale was considered appropriate for the assessment of study quality ( 21 ) and established a value ladder, with a score of 5 for low-quality studies, 6-7 for medium-quality studies, and 8-9 for high-quality studies.

### Data analysis

Multivariable ORs and 95% CIs from each study were merged to assess the predictive effect of factors for post-PN trifecta achievement. Only the factors reported by more than two studies were included in the meta-analyses. The Cochrane Q p value and I2 statistic were used to determine the heterogeneity between reports. This was deemed to be significant when p <0.05 or I2> was 50%, and a random-effect model was used to combine the results. Or else, a fixed-effect model was used. To assess publication bias (only for comparisons that include most studies), we examined funnel plots and performed sensitivity analyses of these comparisons. P <0.05 was considered statistically significant. All statistical analyses were conducted using Review Manager 5.3 (Cochrane Collaboration, Oxford, UK) and Stata 12.0 software (StatCorp, College Station, TX, USA).

## RESULTS

### Included studies

**Figure 1:**
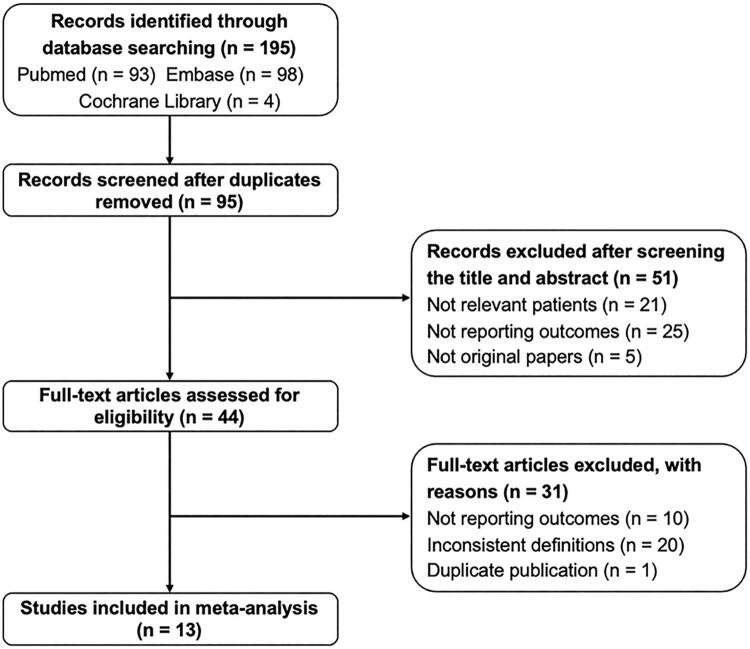
Flow diagram detailing the search strategy and identification of studies included in data synthesis.

According to the flowchart of literature searching ( Figure-1 ), 44 studies were selected for detailed evaluation. Of them, 10 were excluded due to not reporting outcomes, 20 describing inconsistent definitions of trifecta were excluded, and 1 was a duplicate publication. Finally, 13 studies meeting the inclusion criteria were included ( 16 - 20 , 22 - 29 ).

### Baseline characteristics of studies

Eight studies relied on data from multi-institutional database, five studies analyzed patients in single center. Two studies prospectively collected data, and the rest studied retrospectively collected data. In terms of country, 3 were from Japan, 3 from Italy, 2 from France, 2 from Germany, 2 from USA, 1 from Korea. The median sample size was 285 (60-2392). The median or mean age ranged from 49.5 to 63.2 years. Most PN were performed in minimally invasive approach (laparoscopic or robot-assisted). The rate of trifecta achievement ranged from 43.3% to 78.6% ( Table-1A ). The detailed variables in multivariable analysis are presented in Table-2 , most of them were patients features, tumor characteristics, and surgical variables. Six were medium-quality (score 6-7) studies, seven were high-quality (score 8) studies, the detailed risk of bias for each study is presented in supplementary Table-S1 . The other characteristics and perioperative outcomes are detailed in supplementary Table-1B .

**Table 1A:** Baseline characteristics of included studies.

First author	Year	Design	Patient population	Study period	Country	Sample size	Age (years)	Procedure		T stage	Trifecta (%)
Furukawa et al. ( 22 )	2020	Retro	Multi-institution	2011-2016	Japan	804	63 (55-70)	RAPN		pT1a-T3a	62.1
Takeda et al. ( 23 )	2020	Retro	Single institution	2006-2016	Japan	66	54.5 Mean	LPN		cT1a	55
Peyronnet et al. ( 16 )	2018	Retro	Multi-institution	2009-2015	France	1099	60.3 Mean	RAPN		-	75.2
Khene et al. ( 17 )	2018	Retro	Multi-institution	2010-2016	France	500	59 (51-67)	RAPN		pT1-T3a	70.4
Harke et al. ( 18 )	2018	Retro	Multi-institution	2008-2016	Germany	140	-	OPN/RAPN		-	OPN: 68.4; RAPN: 75.0
Castellucci et al. ( 19 )	2018	Retro	Single institution	2013-2016	Italy	123	63.2±13.6	RAPN		-	64.2
Paulucci et al. ( 24 )	2017	Retro	Multi-institution	2008-2016	USA	960	61 (51-69)	RAPN		-	72.2
Lebentrau et al. ( 25 )	2017	Retro	Single institution	2006-2013	Germany	124	-	OPN		cT1	69.4
Porpiglia et al. ( 26 )	2016	Pro	Multi-institution	2009-2012	Italy	285	60.3±14.3	OPN/LPN/RAPN		cT1b	OPN: 62.4; LPN: 63.2; RAPN: 69.5
Kim et al. ( 27 )	2016	Retro	Single institution	2006-2015	Korea	60	49.5 (39.8-62)	RAPN		cT1b	43.3
Zargar et al. ( 20 )	2015	Retro	Multi-institution	2004-2013	USA	1831	-	LPN/RAPN		cT1a	LPN: 33.0; RAPN: 70.0
Osaka et al. ( 28 )	2015	Retro	Single institution	2007-2012	Japan	63	57.9±10.2	LPN		cT1a	61.9
Minervini et al. ( 29 )	2014	Pro	Multi-institution	2009-2011	Italy	450	62.7 Mean	OPN/LPN		cT1a	OPN: 78.6; LPN: 74.3

**Retro** = retrospective; **Pro** = prospective; **RAPN** = robot-assisted partial nephrectomy; **LPN** = laparoscopic partial nephrectomy; **OPN** = open partial nephrectomy.

**Table 1B:** The other characteristics and perioperative outcomes for included studies.

First author	Year	Gender (male/female)	Median BMI (kg/m2)	Median tumor size (cm)	Median nephrometry score	Total complication (n)	Median OT (min)	Median WIT (min)	Median EBL (mL)	Median LOS (d)	PSM (n)
Furukawa et al. ( 22 )	2020	584/220	-	2.6	7 R	132 (Clavien≥III: 74)	234	21	30	9	8
Takeda et al. ( 23 )	2020	55/11	-	-	-	8 (I: 0, II: 3, III: 5)	-	-	-	-	0
Peyronnet et al. ( 16 )	2018	712/387	-	-	-	162 (Clavien≥III: 60)	-	-	-	-	56
Khene et al. ( 17 )	2018	297/203	27	3.3	7 R	125 (Clavien≥III: 49)	160	15	250	3	19
Harke et al. ( 18 )	2018	90/50	-	-	11 P	30 (Clavien≥III: 16)	-	-	-	-	2
Castellucci et al. ( 19 )	2018	70/53	27	-	-	23 (II: 15, III: 7, IV:1)	115	-	205	-	14
Paulucci et al. ( 24 )	2017	568/392	29.3	3	7 R	115 (Clavien≥III: 33)	179	16	100	1	38
Lebentrau et al. ( 25 )	2017		-	-	-	-	-	-	-	-	8
Porpiglia et al. ( 26 )	2016	171/114	25.9	5	-	31 (II: 17, III: 8)	135	16	200	-	9
Kim et al. ( 27 )	2016	33/27	24.7	5	9 R	9 (II: 7, III: 2, IV:0)	165.5	-	425	-	4
Zargar et al. ( 20 )	2015	1097/734	30	-	-	359	-	-	-	-	100
Osaka et al. (89)	2015	50/13	24.7	24	6 R	4	177	21	87	-	4
Minervini et al. ( 29 )	2014	179/101	-	2.5	-	46	-	-	-	-	-

**BMI** = body mass index; **OT** = operative time; **WIT** = warm ischemia time; **EBL** = estimated blood loss; **LOS** = length of hospital stay; **PSM** = positive surgical margin; **R** = RENAL; P = PADUA.

**Table 2:** Included variables in multivariable analysis and study quality.

Author	Year	Variables included in multivariable analysis	NOS score
Furukawa et al. ( 22 )	2020	tumor size, OT, EBL, RENAL score, N score component, hilar location	8
Takeda et al. ( 23 )	2020	tumor size	6
Peyronnet et al. ( 16 )	2018	tumor size, RENAL score, surgeon experience, surgeon volume, hospital volume	8
Khene et al. ( 17 )	2018	age, CCI, ECOG, tumor size, RENAL score, MAP score	8
Harke et al. ( 18 )	2018	age, BMI, CCI, tumor size, solitary kidney, PADUA score, OPN vs RAPN, experience	8
Castellucci et al. ( 19 )	2018	age, symptoms, tumor size, PADUA score, preoperative eGFR, EBL, OT	7
Paulucci et al. ( 24 )	2017	surgeon experience, tumor size	7
Lebentrau et al. ( 25 )	2017	age, sex, BMI, eGFR, ASA, PADUA score, surgical experience	7
Porpiglia et al. ( 26 )	2016	tumor growth pattern, EBL, centers	8
Kim et al. ( 27 )	2016	tumor size, OT, EBL	7
Zargar et al. ( 20 )	2015	RAPN vs LPN, tumor size, RENAL score, EBL, OT	8
Osaka et al. ( 28 )	2015	preoperative eGFR, tumor size, nearness of UCS, Surgeon’s learning curve	7
Minervini et al. ( 29 )	2014	age, tumor size, indication	8

**OT** = operative time; **EBL** = estimated blood loss; **CCI** = charlson’s comorbidity index; **ECOG** = Eastern Cooperative Oncology Group; **BMI** = body mass index; **OPN** = open partial nephrectomy; **RAPN** = robot-assisted partial nephrectomy; **eGFR** = estimated glomerular ﬁltration rate; **ASA** = American Society of Anesthesiologists; **LPN** = laparoscopic partial nephrectomy; **UCS** = urinary collecting system.

### Predictors of trifecta achievement

**Figure 2:**
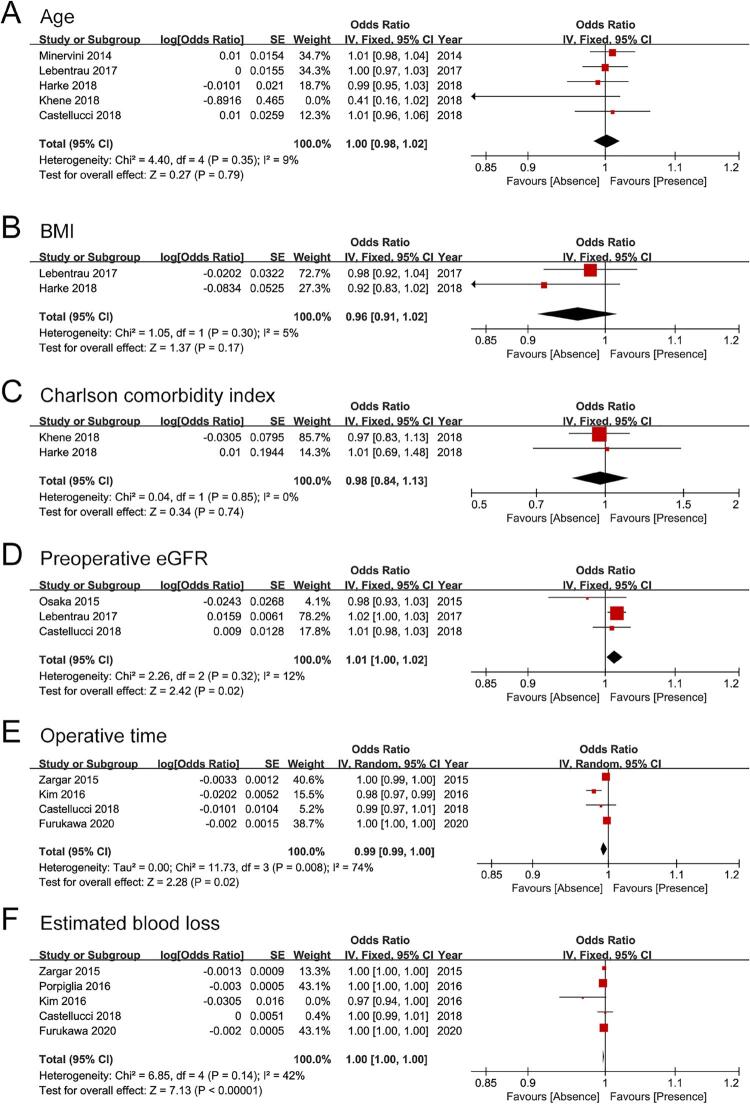
Forest plots for predictors of trifecta achievement. The predictors included (A) age, (B) body mass index, (C) Charlson comorbidity index, (D) preoperative estimated glomerular ﬁltration rate, (E) operative time, (F) estimated blood loss.

Most predictive factors were patients features, surgical variables, and tumor characteristics. Patients features were analyzed as continuous variables. Since no significant heterogeneity was identified (I2=0%-12%, P=0.30-0.85), the fixed-effect model was used. A pooled analysis of ORs proved age (OR: 1.00, 95% CI: 0.98, 1.02, P=0.79), body mass index (OR: 0.96, 95% CI: 0.91, 1.02, P=0.17), Charlson comorbidity index (OR: 0.98, 95% CI: 0.84, 1.13, P=0.74) weren’t independent predictive factors for trifecta achievement ( Figures 2 A-C ). The merged results showed that preoperative eGFR (OR: 1.01, 95% CI: 1.00, 1.02, P=0.02) was independently associated with trifecta achievement, but the predictive effect was minor ( Figure-2D ).

Since significant heterogeneity was identified (I2=74%, P=0.008), the random-effect model was used for operative time. Since no significant heterogeneity was identified (I2=42%, P=0.14), the fixed-effect model was used for estimated blood loss. A pooled analysis of ORs demonstrated operative time (OR: 0.99, 95% CI: 0.99, 1.00, P=0.02) and estimated blood loss (OR: 1.00, 95% CI: 1.00, 1.00, P <0.001) were independently associated with trifecta achievement, but the predictive effect was minor ( Figures 2E and F ).

**Figure 3:**
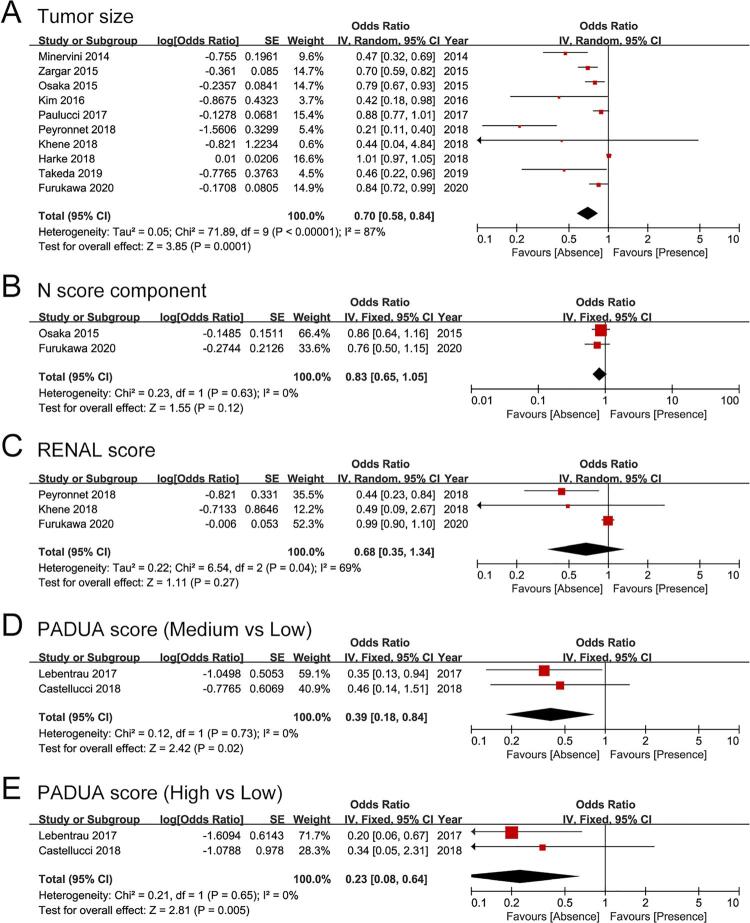
Forest plots for predictors of trifecta achievement. The predictors included (A) tumor size, (B) N score component, (C) RENAL score, (D) PADUA score (medium vs. low), (E) PADUA score (high vs. low).

Tumor characteristics included tumor size, N score component, RENAL score, and PADUA score. Due to significant heterogeneity, the random-effect model was used for tumor size and RENAL score, the fixed-effect model was used for other meta-analyses. Pooled analysis of ORs demonstrated tumor size (OR: 0.70, 95% CI: 0.58, 0.84, P <0.001), medium (OR: 0.39, 95% CI: 0.18, 0.84, P=0.02) and high PADUA score (OR: 0.23, 95% CI: 0.08, 0.64, P=0.005) were independently associated with trifecta achievement ( Figures 3A , D and E ). N score component (OR: 0.83, 95% CI: 0.65, 1.05, P=0.12) and RENAL score (OR: 0.68, 95% CI: 0.35, 1.34, P=0.27) weren’t independent predictive factors for trifecta achievement ( Figures 3 B and C ).

### Bias assessment

**Figure 4:**
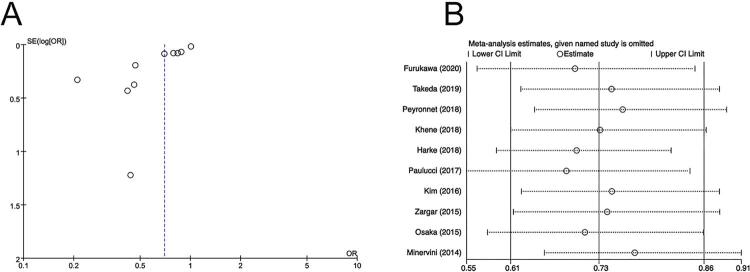
(A) Funnel plot to assess publication bias for tumor size, (B) sensitivity analysis for tumor size.

Given the inadequate studies, publication bias checking and sensitivity analysis were only performed for tumor size. The funnel plot seemed to be asymmetric ( Figure-4A ), and Egger’s test identified significant difference (P=0.001). Sensitivity analysis confirmed the stability of results ( Figure-4 B).

## DISCUSSION

A comprehensive outcome measure, the trifecta achievement (i.e., negative surgical margins, warm ischemia time <25 minutes, no complications), has been recommended as a measure of postoperative surgical quality for PN ( 24 , 26 , 28 , 29 ). Some perioperative parameters including patient features, tumor characteristics, and surgical variables were hypothesized to be associated with the trifecta achievement of PN. We firstly assessed the predictive factors of trifecta achievement for patients undergoing PN with the method of systematic review and meta-analysis. The present study included 7.066 patients, and the rate of trifecta achievement ranged from 43.3% to 78.6%. High variability was found regarding the rate of trifecta achievement may due to the differences in patient condition, tumor size and stage, surgical approach, and so on.

More than thirty studies have reported predictive factors for trifecta achievement, however, different definitions of trifecta achievement were described. Trifecta achievement was consisted of three aspects, namely surgical margin, renal function preservation, and perioperative complication. The inconsistency lies mainly in the latter two aspects. The most common definition was adopted, specifically negative surgical margins, warm ischemia time <25 minutes, and no complications. Finally, 13 studies meeting the inclusion criteria were included ( 16 - 20 , 22 - 29 ). The detailed variables in multivariable analysis are presented in Table- 2, most of them were patients features, tumor characteristics, and surgical variables. For the same variables, different forms of data were used in different studies, and the most common data type was chosen. Based on the results from multivariable analyses, several independent predictors have been identified.

Patients features including age, body mass index, Charlson comorbidity index, and preoperative eGFR were analyzed as continuous variables. Only preoperative eGFR was found to be independently associated with trifecta achievement. However, the predictive effect was minor, the odd ratio was 1.01 (1.00-1.02). Moreover, a recent study based on 790 patients treated with laparoscopic PN found that preoperative eGFR (OR: 1.01, 95% CI: 1.00, 1.02) was associated with an increased probability of pentafecta achievement ( 30 ). These results indicated that preoperative eGFR had a limited effect on postoperative outcomes. Surgical variables including operative time and estimated blood loss were analyzed as continuous variables. Though they were found to be independently associated with trifecta achievement, the predictive effect was minor, the odd ratios were 0.99 (0.99-1.00) and 1.00 (1.00-1.00). Moreover, these two variables were related to surgery, and only can be obtained after surgery, their predictive value was limited.

Tumor characteristics including tumor size, N score component, RENAL score, and PADUA score were analyzed. The variable tumor size has been most studied and reported in the included literatures. Merged data showed that tumor size (OR: 0.70, 95% CI: 0.58, 0.84) was associated with a decreased probability of trifecta achievement. This result is reasonable because tumor size obviously affects the two components (renal function, perioperative complication) of trifecta achievement. Reynolds et al. ( 31 ) have compared perioperative and functional outcomes for patients with clinical T1a and T1b renal tumors undergoing robot-assisted PN. They found that clinical T1a tumors were correlated with shorter warm ischemia time, lower rate of perioperative complications. Similarly, in the setting of robot-assisted PN, Delto et al. ( 32 ) have compared perioperative outcomes for patients with clinical T1a, T1b, and T2a renal tumors. They found that clinical T2a renal tumors were associated with a 7% increase in warm ischemia time, a 3.93 higher odds of acute kidney injury compared to T1a renal tumors. Both the two studies didn’t identify significant difference in surgical margins among different clinical stage renal tumors. Due to the significant effect of tumor size on ischemia time and perioperative complication, Castellucci et al. ( 33 ) have reported that patients with renal masses ≥4cm achieved an obviously lower rate of trifecta achievement (44.7% vs. 72.9%) than those with renal masses <4cm.

In terms of anatomic scoring systems, renal tumors with medium (OR: 0.39, 95% CI: 0.18, 0.84) and high PADUA score (OR: 0.23, 95% CI: 0.08, 0.64) were associated with decreased probability of trifecta achievement when compared with those with low PADUA score. These results seemed to be reasonable, more complex tumors may experience more unfavorable perioperative outcomes. However, no significant difference was identified for N score component and RENAL score. The possible reasons included limited studies have reported these results, and these two variables were analyzed in continuous variable which underestimate the differences.

Though the present study stands for the first systematic review and meta-analysis about the predictive factors for trifecta achievement in patients undergoing partial nephrectomy, several limitations need to be addressed. First, all included studies were retrospectively designed or database based, and therefore inherent biases were included. Some studies were of moderate quality and cannot be comparable for each related variable. Hence, we just analyzed the results from multivariable analyses which adjusted the confounding factors. Second, more than thirty studies have reported predictive factors for trifecta achievement, however, different definitions of trifecta achievement were described. The most common definition was adopted, then 13 studies were included. Some endpoints were reported by limited studies and analyzed in different data type, the pooled results for theses endpoints should been verified by further studies. Moreover, due to the inadequate studies, some important variables such as surgical approach have not been analyzed in our study. Third, there were significant heterogeneity among studies for some endpoints, such as tumor size, operative time. The publication bias checking identified a potential publication bias for tumor size. Hence, these results might be interpreted with caution.

## CONCLUSIONS

Trifecta achievement provides a definition of an ideal surgical outcome for patients undergoing partial nephrectomy. Larger tumor size, medium and high PADUA score are associated with decreased probability of trifecta achievement. After verifying by further high-quality studies, these variables can be incorporated into tools to predict probability of trifecta achievement during clinical practice.

## APPENDIX 1


