# Supplementary material for: Trifecta achievement in patients undergoing partial nephrectomy: a systematic review and meta-analysis of predictive factors
Source: Int Braz J Urol. 2021 Apr 20;48(4):625–35. doi: 10.1590/S1677-5538.IBJU.2021.0095 (PMC9306373; doi:10.1590/S1677-5538.IBJU.2021.0095)
Supplement: Supplementary file 1 [file 1677-6119-ibju-48-04-0625-suppl01.pdf]

APPENDIX 1

Table S1 - Risk of bias for each included study.

Study	Year	Selection				Comparability	Outcome			Overall
		Representativeness of exposed cohort	Selection of nonexposed	Ascertainment of exposure	Outcome not present at start		Assessment of outcome	Adequate follow-up length	Adequacy of follow-up	
Furukawa et al. (22)	2020	1	1	1	1	1	1	1	1	8
Takeda et al. (23)	2020	0	1	1	1	1	0	1	1	6
Peyronnet et al. (16)	2018	1	1	1	1	1	1	1	1	8
Khene et al. (17)	2018	1	1	1	1	1	1	1	1	8
Harke et al. (18)	2018	1	1	1	1	1	1	1	1	8
Castellucci et al. (19)	2018	0	1	1	1	1	1	1	1	7
Paulucci et al. (24)	2017	1	1	1	1	1	0	1	1	7
Lebentrau et al. (25)	2017	0	1	1	1	1	1	1	1	7
Porpiglia et al. (26)	2016	1	1	1	1	1	1	1	1	8
Kim et al. (27)	2016	0	1	1	1	1	1	1	1	7
Zargar et al. (20)	2015	1	1	1	1	1	1	1	1	8
Osaka et al. (28)	2015	0	1	1	1	1	1	1	1	7
Minervini et al. (29)	2014	1	1	1	1	1	1	1	1	8
